# Soldering Coarse Metals

**Published:** 1883-05

**Authors:** 


SOLDERING COARSE METALS.—The following method of soldering without the use of a soldering iron is given in the *Techniker* :

The parts to be joined are made to fit accurately, either by filing or on a lathe. The surfaces are moistened with the soldering fluid, a smooth piece of tinfoil laid on, and the pieces pressed together and tightly wired. The article is then heated over the fire by means of a lamp until the tinfoil melts. In this way two pieces of brass can be soldered together so nicely that the joint can scarcely be found.

With good soft solder, nearly all kinds of soldering can be done over a lamp without the use of a "bit." If several pieces have to be soldered on the same piece, it is well to use solder of unlike fusibility. If the first piece is soldered with fine solder, composed of two parts of lead, one of tin, and two of bismuth, there is no danger of its melting when another place near it is soldered with bismuth solder, made of four parts of lead, four of tin, and one of bismuth, for their melting points differ so much that the former will not melt when the latter does. Many solders do not form any malleable compounds.

In soldering together brass, copper, or iron, hard solder must be employed; for example, a solder made of equal parts of brass and silver. For iron, copper, or brass of high melting point, a good solder is obtained by rolling a silver coin out thin; for it furnishes a tenacious compound, and one that is not too expensive, since silver stretches out well. Borax is the best flux for hard soldering. It dissolves the oxides which form on the surface of the metal, and protects it from further oxidation, so that solder comes into actual contact with the surfaces of the metal. For soft soldering, the well-known fluid, made by saturating equal parts of water and hydro-chloric acid with zinc, is to be used. In using common solder rosin is the cheapest and best flux. It also has this advantage, that it does not rust the article that it is used on.

